# Supplementary material for: A multi-factor dynamic time series measure for stock correlation analysis
Source: PLoS One. 2025 Dec 15;20(12):e0336173. doi: 10.1371/journal.pone.0336173 (PMC12704864; doi:10.1371/journal.pone.0336173)
Supplement: Attachment — Submitted filename: Responses to Reviewer .docx [file pone.0336173.s002.docx]

**Response to Reviewers**

**Manuscript ID:** PONE-D-25-44140

**Title:** A Multi-Factor Dynamic Time Series Measure for Stock Correlation Analysis

Dear Dr. Najmul Hasan and Reviewers,

We sincerely thank the editors and reviewers for their valuable time and insightful comments on our manuscript. These comments have significantly helped us improve the quality and clarity of our work. We have carefully addressed all the points raised. Below is our point-by-point response to the comments from Reviewer 1 and Reviewer 2.

Changes in the manuscript have been highlighted in yellow in the revised manuscript file with track changes.

**Responses to Reviewer 1:**

**Comment 1:** In the Introduction section, the authors provided extensive information on time series analysis methods such as TLE, PPC, LCSS and DTW to emphasise the innovativeness of their proposed Multi-Factor Dynamic Temporal Similarity Measure (MFDTSM) method. However, the Introduction Section lacks information on modern time series analysis methods using neural networks.

**Response:** We sincerely thank the reviewer for this excellent suggestion. We agree that discussing modern neural network-based approaches is crucial for positioning our work within the current research landscape. We have now added a new paragraph in the Introduction section that introduces prevalent deep learning methods like LSTM, GRU, and Transformers for time series analysis.

The added text acknowledges their strengths in capturing complex temporal dependencies while also explaining our methodological choice by highlighting their limitations relative to the goals of our study, particularly regarding interpretability of factor-specific influences and explicit modeling of dynamic phase shifts in time-lag effects. This addition provides a more comprehensive background and better underscores the unique niche our MFDTSM method aims to fill.

**Action Taken:** Added a new paragraph on modern neural network methods in the Introduction. And the newly added paragraph is as follows:

In recent years, deep learning approaches, particularly neural network-based models such as Long Short-Term Memory (LSTM) , Gated Recurrent Units (GRU) , and Transformers , have also been widely adopted for time series analysis due to their ability to capture complex temporal dependencies and nonlinear patterns. For instance, LSTMs have been successfully applied in stock price prediction and portfolio optimization, while attention mechanisms enhance the model’s focus on salient time steps. However, these methods often require large amounts of data and computational resources, and their black-box nature can limit interpretability in factor-specific analysis. Although neural networks excel in pattern recognition, they are less focused on explicitly modeling factor influence and dynamic phase shifts in time-lag effects, which are central to our proposed MFDTSM framework.

**Comment 2:** Through their research, the authors demonstrated the high efficiency of the MFDTSM method. However, they paid little attention to analysing how sensitive the results were to the choice of XGBoost hyperparameters and clustering parameters.

**Response:** We thank the reviewer for raising this important point regarding the sensitivity and robustness of our model. To address this, we have expanded the Experimental Setup section with a more detailed explanation of our hyperparameter selection process.

We now explicitly state that key hyperparameters, including the number of clusters (c=5) and the phase change rate coefficient (μ=1), were determined through a grid search and cross-validation process to ensure robustness. We also mention that we employed the Silhouette Score and Elbow Method to evaluate and confirm the optimal choice of the cluster number. Furthermore, we note that although the model performance is relatively stable across slight variations in these parameters, we recommend more advanced methods like Bayesian optimization for future work. This addition demonstrates our careful consideration of parameter tuning and enhances the reproducibility of our study.

**Action Taken:** Enhanced the description of hyperparameter selection in the Experimental Setup section. And the newly added paragraph is as follows:

The selection of hyperparameters, including the number of clusters c = 5 and the phase change rate coefficient μ = 1, was determined through a grid search and cross-validation process to ensure robustness. We evaluated clustering performance using the Silhouette Score and Elbow Method, which indicated that c = 5 optimally balances intra-cluster cohesion and inter-cluster separation. Similarly, μ was tuned in the range [0.5, 2.0] to minimize prediction error in a validation set. Although the model performance is relatively stable across slight variations in these parameters, future work could incorporate Bayesian optimization for more efficient hyperparameter tuning.

**Comment 3:** The authors provided little information on how they selected the 12 measurement factors. There is also no information on how the selection of factors affects the stability of the model. This information should be added to the Conclusions section.

**Response:** We appreciate the reviewer for this critical feedback. We have now added a comprehensive paragraph in the Conclusions section to address both parts of this comment.

Firstly, we explain that the selection of the 12 factors (e.g., price, turnover rate, moving averages) was based on two criteria: (1) their established importance and consensus in existing financial literature, and (2) a preliminary feature importance analysis conducted using tree-based models.

Secondly, and more importantly, we directly address the model's stability concerning factor selection. We describe that we conducted a sensitivity analysis by alternately excluding one factor at a time and observing the fluctuation in the model's similarity accuracy. The results showed that the model maintains robust performance with a minimal variation in accuracy (±2.3% in Mean Absolute Percentage Error, MAPE), indicating that MFDTSM is not overly reliant on any single factor and is generally stable under factor variations. This new analysis provides concrete evidence for the robustness of our method.

**Action Taken:** Added a new paragraph detailing factor selection and stability analysis in the Conclusions section. And the newly added paragraph is as follows:

Furthermore, the selection of the 12 stock factors (e.g., price, turnover rate, moving averages) was based on both the established financial research consensus and preliminary feature importance analysis using tree-based models. These factors are widely recognized as key indicators of stock performance and market behavior. To assess the stability of MFDTSM with respect to factor selection, we conducted sensitivity analyses by alternately excluding one factor at a time. The results showed that the model maintains robust performance with minimal fluctuation in similarity accuracy (±2.3% in Mean Absolute Percentage Error, MAPE), indicating that MFDTSM is not overly reliant on any single factor and is generally stable under factor variation.

Once again, we express our deepest gratitude to the reviewers and editors for their constructive comments, which have greatly improved our manuscript. We look forward to the positive consideration of our revised paper.

**Responses to Reviewer 2:**

**Comment 1:** It would be more intuitive if a list of the 102 stocks could be provided.

Response: We sincerely thank the reviewer for this valuable suggestion. To enhance the transparency and reproducibility of our study, we have now included a complete list of the 102 stocks used in our analysis as supplementary material. The list details the stock codes, stock names, primary industries (L1), and secondary industries (L2) based on the Shenwan Industry Classification Standard. Specifically, the dataset comprises 73 stocks from the Communication industry and 29 stocks from the Financial industry, covering diverse sub-sectors such as Communication Equipment, Communication Operations, Computer Applications, Bank, Real Estate, and others. This addition allows readers to better understand the dataset composition and facilitates further validation and replication of our study.

**Action Taken:** We have added a supplementary file named “S1_Table.xlsx” containing the complete stock list.

| **Stock Code** | **Stock Name** | **Industry (L1)** | **Industry (L2)** |
| --- | --- | --- | --- |
| 000063 | ZTE Corporation | Communication | Communication Equipment |
| 000547 | Aerospace Development | Communication | Communication Equipment |
| 000561 | Fenghuo Electronics | Communication | Communication Equipment |
| 000586 | Huiyuan Communication | Communication | Communication Equipment |
| 000665 | Hubei Broadcasting | Communication | Communication Operations |
| 000701 | Xiamen Xinda | Communication | Communication Operations |
| 000725 | BOE Technology Group | Communication | Optoelectronic Devices |
| 000748 | Great Wall Information | Communication | Computer Applications |
| 000777 | Zhonghe Technology | Communication | Communication Equipment |
| 000839 | CITIC Guoan | Communication | Communication Operations |
| 000876 | New Hope | Communication | Communication Operations |
| 000938 | Unisplendour Corporation | Communication | Computer Equipment |
| 000988 | Huagong Tech | Communication | Communication Equipment |
| 002049 | Unigroup Guoxin | Communication | Integrated Circuits |
| 002089 | Sunsea Telecommunications | Communication | Communication Equipment |
| 002093 | Guomai Technologies | Communication | Communication Operations |
| 002104 | Hengbao Co., Ltd. | Communication | Computer Applications |
| 002115 | Sunwave Communications | Communication | Communication Equipment |
| 002151 | BDStar Navigation | Communication | Communication Equipment |
| 002161 | Yuanwanggu | Communication | Communication Equipment |
| 002194 | Wuhan Fangu | Communication | Communication Equipment |
| 002212 | Topsec Technologies | Communication | Computer Applications |
| 002231 | Allwin Telecommunications | Communication | Communication Equipment |
| 002261 | TOWIN Information | Communication | Computer Applications |
| 002281 | Accelink Technologies | Communication | Communication Equipment |
| 002296 | Splendor Science & Technology | Communication | Computer Applications |
| 002313 | Sunsea IoT | Communication | Communication Equipment |
| 002316 | Yalian Development | Communication | Computer Applications |
| 002396 | Star-net Communication | Communication | Communication Equipment |
| 002417 | Shennan Circuits | Communication | Computer Applications |
| 002465 | Haige Communications | Communication | Communication Equipment |
| 002467 | 263 Network | Communication | Communication Operations |
| 002544 | P&T Technology | Communication | Communication Equipment |
| 300050 | Dingli Communications | Communication | Communication Operations |
| 300051 | 35 Internet | Communication | Computer Applications |
| 300052 | Zhongqingbao | Communication | Internet Services |
| 300074 | Huaping Stock | Communication | Computer Applications |
| 300098 | Gaoxin Development | Communication | Communication Equipment |
| 300134 | Taffull Technology | Communication | Communication Equipment |
| 300136 | Sunway Communication | Communication | Communication Equipment |
| 300166 | Beyondsoft | Communication | Computer Applications |
| 300168 | Wonders Information | Communication | Computer Applications |
| 300177 | Zhonghaida | Communication | Communication Equipment |
| 300209 | Tianze Information | Communication | Computer Applications |
| 300226 | Shanghai Steel Union | Communication | Internet Services |
| 300245 | Tianji Technology | Communication | Computer Applications |
| 300250 | Chuling Information | Communication | Communication Equipment |
| 300252 | Jinxinnuo | Communication | Communication Equipment |
| 300264 | Jachung Vision | Communication | Computer Applications |
| 300288 | Langma Information | Communication | Computer Applications |
| 300290 | Rongke Technology | Communication | Computer Applications |
| 600050 | China Unicom | Communication | Communication Operations |
| 600105 | Yongding Stock | Communication | Communication Equipment |
| 600130 | Bird Corporation | Communication | Communication Equipment |
| 600198 | Datang Telecom | Communication | Communication Equipment |
| 600345 | Changjiang Communication | Communication | Communication Equipment |
| 600485 | Xinwei Group | Communication | Communication Operations |
| 600487 | Hengtong Optic-Electric | Communication | Communication Equipment |
| 600498 | FiberHome Telecommunications | Communication | Communication Equipment |
| 600522 | Zhongtian Technology | Communication | Communication Equipment |
| 600536 | China National Software | Communication | Computer Applications |
| 600570 | Hundsun Technologies | Communication | Computer Applications |
| 600588 | Yonyou Network | Communication | Computer Applications |
| 600637 | Oriental Pearl | Communication | Communication Operations |
| 600640 | Guomai Culture | Communication | Communication Operations |
| 600776 | Eastern Communications | Communication | Communication Equipment |
| 600797 | Insigma Technology | Communication | Computer Applications |
| 600804 | Dr. Peng Telecom | Communication | Communication Operations |
| 600845 | Baosight Software | Communication | Computer Applications |
| 600850 | East China Computer | Communication | Computer Applications |
| 600980 | Beikang Technology | Communication | Communication Equipment |
| 603000 | People.cn | Communication | Internet Services |
| 603160 | Goodix Technology | Communication | Integrated Circuits |
| 000001 | Ping An Bank | Financial | Bank |
| 000002 | Vanke A | Financial | Real Estate |
| 000006 | Shenzhen Zhenye A | Financial | Real Estate |
| 000012 | CSG A | Financial | Real Estate |
| 000046 | Oceanwide Holdings | Financial | Real Estate |
| 000069 | OCT Group A | Financial | Real Estate |
| 000402 | Financial Street | Financial | Real Estate |
| 000415 | Bohai Leasing | Financial | Other Financial |
| 000416 | Minsheng Holdings | Financial | Other Financial |
| 000417 | Hefei Department Store | Financial | Other Financial |
| 000419 | Tongcheng Holdings | Financial | Other Financial |
| 000420 | Jilin Chemical Fiber | Financial | Other Financial |
| 000421 | Nanjing Public Utility | Financial | Other Financial |
| 000423 | Dong-E-E-Jiao | Financial | Other Financial |
| 000425 | XCMG Construction Machinery | Financial | Other Financial |
| 000426 | Xingye Mining | Financial | Other Financial |
| 000428 | Huatian Hotel | Financial | Other Financial |
| 000429 | Guangdong Expressway A | Financial | Other Financial |
| 000430 | Zhangjiajie Tourism | Financial | Other Financial |
| 000488 | Chenming Paper | Financial | Other Financial |
| 000498 | Shandong Road & Bridge | Financial | Other Financial |
| 000501 | Wushang Group A | Financial | Other Financial |
| 000503 | Guoxin Health | Financial | Other Financial |
| 000506 | Zhongrun Resources | Financial | Other Financial |
| 000507 | Zhuhai Port | Financial | Other Financial |
| 000509 | Huasu Holdings | Financial | Other Financial |
| 000510 | Xin Jinlu | Financial | Other Financial |
| 000513 | Livzon Group | Financial | Other Financial |
| 000514 | Yujian Development | Financial | Real Estate |
| 000063 | ZTE Corporation | Communication | Communication Equipment |
| 000547 | Aerospace Development | Communication | Communication Equipment |
| 000561 | Fenghuo Electronics | Communication | Communication Equipment |
| 000586 | Huiyuan Communication | Communication | Communication Equipment |
| 000665 | Hubei Broadcasting | Communication | Communication Operations |
| 000701 | Xiamen Xinda | Communication | Communication Operations |
| 000725 | BOE Technology Group | Communication | Optoelectronic Devices |
| 000748 | Great Wall Information | Communication | Computer Applications |
| 000777 | Zhonghe Technology | Communication | Communication Equipment |
| 000839 | CITIC Guoan | Communication | Communication Operations |
| 000876 | New Hope | Communication | Communication Operations |
| 000938 | Unisplendour Corporation | Communication | Computer Equipment |
| 000988 | Huagong Tech | Communication | Communication Equipment |
| 002049 | Unigroup Guoxin | Communication | Integrated Circuits |
| 002089 | Sunsea Telecommunications | Communication | Communication Equipment |
| 002093 | Guomai Technologies | Communication | Communication Operations |
| 002104 | Hengbao Co., Ltd. | Communication | Computer Applications |
| 002115 | Sunwave Communications | Communication | Communication Equipment |
| 002151 | BDStar Navigation | Communication | Communication Equipment |
| 002161 | Yuanwanggu | Communication | Communication Equipment |

**Comment 2:** It is recommended to label the meanings of different types in Figure 3 to make it easier for readers to understand the content of the figure.

**Response:** We sincerely thank the reviewer for this valuable and constructive suggestion. We agree that clarifying the elements of Figure 3 is crucial for helping readers intuitively understand the distribution and comparison of prediction errors across different methods. In response to this comment, we have implemented the following comprehensive revisions to the figure and its corresponding description in the text to significantly enhance its clarity and interpretability.

**Action Taken:**

1. **Enhanced Textual Explanation:** In the "Results Analysis and Summary" section, we have added a detailed paragraph explaining the statistical elements represented in the box plots. This addition explicitly describes that:

·The boxes visualize the interquartile range (IQR), which contains the middle 50% of the data (from the 25th to the 75th percentiles).

·The horizontal line inside each box denotes the median value.

·The whiskers extend to show the range of the data within 1.5 times the IQR from the quartiles.

·Individual points beyond the whiskers are plotted as outliers.

This explanation provides readers with the necessary context to correctly interpret the visual information in the figure.

2. **Revised Subfigure Captions:** We have thoroughly updated all individual subfigure captions to be self-explanatory. Each caption now explicitly states:

·The specific evaluation metric being displayed (MAE or MAPE).

·The full name of the metric in parentheses (e.g., "Mean Absolute Error").

·The machine learning model under which the performance was evaluated (LR, RF, or SVM). For instance, the original caption "MAE performance under LR" was revised to "MAE Distribution of Different Similarity Measures under LR Model (MAE: Mean Absolute Error)". This change ensures that readers can immediately grasp the content of each subfigure without ambiguity.

3. **Updated Overall Figure Caption:** The main caption for the composite figure has been updated to "Box Plot of Evaluation Parameters (MAE, MAPE) for Different Similarity Measures in Machine Learning Models (LR, RF, SVM)". This provides a clear and comprehensive overview of the figure's purpose, specifying the parameters, methods, and models being compared.

Collectively, these revisions ensure that the meaning of different graphical elements and the content of each subfigure are immediately clear to the reader, thereby facilitating an intuitive and accurate comparison of the prediction error distributions and the robustness of the different similarity measurement methods.

Sincerely,

Jun Ma, Ph.D.

Corresponding Author

On behalf of all co-authors.
